# Vaccination of immune compromised children—an overview for physicians

**DOI:** 10.1007/s00431-021-03997-1

**Published:** 2021-03-05

**Authors:** Laure F. Pittet, Klara M. Posfay-Barbe

**Affiliations:** 1 Infectious Diseases Unit, Royal Children’s Hospital Melbourne, Parkville, Victoria Australia; 2 Unit of Pediatric Infectious Diseases, Division of General Pediatrics, Department of Pediatrics, Gynecology & Obstetrics, Children’s Hospital, University Hospitals of Geneva, 6 Rue Willy Donzé, 1211 Geneva, Switzerland; 3 Faculty of Medicine, University of Geneva, Rue Michel-Servet 1, 1211 Geneva, Switzerland

**Keywords:** Immunosuppression, Immunization, Vaccine-preventable diseases, Paediatrician

## Abstract

Immune compromised children are threatened by a higher risk of infections; some of these are preventable by vaccination. Primary care physicians play a fundamental role in optimising vaccination status. In this narrative review, we present the evidence on vaccine safety and immunogenicity in immune compromised children and discuss in which conditions live-attenuated vaccines can possibly be used. Vaccination schedules differ in some of these conditions, including the use of vaccines with higher antigenic contents (e.g. high-dose hepatitis B vaccine), additional vaccine doses (e.g. 2-dose schedule meningococcal vaccine), more frequent booster doses (e.g. life-long pneumococcal vaccine booster), supplementary vaccines (e.g. meningococcal B vaccine) and use of vaccines beyond the age of usual recommendation (e.g. *Haemophilus influenza* type b vaccine after 5 years of age). Serological monitoring is a useful tool for customizing vaccination schedule in immune compromised children, confirming adequate vaccine response and documenting seroprotection (especially against measles and varicella). Finally, verification of vaccination status of all household members can prevent them being vector of transmission of an infection to the immune compromised children. *Conclusion*: Intensified information strategies are needed to improve trust, rectify perceived risks and improve vaccine acceptability; primary physicians can play a critical role in the latter.

**Table:** 

**What is Known:** • *Physician’s awareness is key to success, since it repeatedly correlates with higher vaccination rates*	
**What is New:** • *The vaccination status of immunocompromised children is rarely up-to-date* • *Knowing the latest vaccine recommendations is challenging, as they differ for each medical condition and change periodically* • *This review summarises the vaccine recommendations for children with compromised immune systems and highlights how paediatricians play a key role in coordinating their application*

**What is Known:**

• *Physician’s awareness is key to success, since it repeatedly correlates with higher vaccination rates*

**What is New:**

• *The vaccination status of immunocompromised children is rarely up-to-date*

• *Knowing the latest vaccine recommendations is challenging, as they differ for each medical condition and change periodically*

• *This review summarises the vaccine recommendations for children with compromised immune systems and highlights how paediatricians play a key role in coordinating their application*

## Introduction

Protecting immune compromised children against infections is challenging and is a problem of growing importance. Indeed, paediatrician are dealing with more and more patients with deficient immune system, as (i) immunosuppressive therapies are increasingly used in various medical conditions and (ii) the life expectancy of patients with these conditions has substantially raised. The quality of life of these children has also improved over the years: they are able to attend school, travel and be active in their community. This inevitably puts them in contact with others and a variety of infectious pathogens. Moreover, the frequent hospital admission and outpatient visits associated with chronic diseases inevitably increase their risk of nosocomial exposure to pathogens.

Vaccination has repeatedly been recognised as one of the most important and most cost-efficient invention in healthcare [1]. Vaccine-preventable diseases occur more frequently and have a worst outcome in immunocompromised individuals. In a retrospective cohort study of nearly 7000 paediatric solid organ recipients, 15.6% were hospitalised for a vaccine-preventable diseases in the first 5 years following transplantation, an 87-fold higher rate compared with the general population [2]. Worst outcomes are well illustrated by the severity of measles infection, which carries a 40% to 70% fatality rate among immunocompromised patients, despite adequate treatment, up to 35-fold higher compared with immunocompetent hosts [3]. Measles and other vaccine-preventable diseases have recently re-emerged in many regions, mostly due to declining vaccine uptake [4, 5]. As herd protection cannot be relied on, prevention of vaccine-preventable diseases in vulnerable population is key.

The aim of this review is to present an overview of the knowledge in the field, provide tables and references that could help primary care physicians when managing immune compromised children. The following questions are addressed: Which fundamental role do primary care physicians play? Who are immune compromised children? Why are their vaccinations status not up-to-date? Are vaccines immunogenic and safe in immune compromised children? Is the vaccination schedule the same than for healthy children? Which additional vaccines are recommended? Why/when should vaccine-preventable diseases serology be monitored? In which situation can live-attenuated vaccine be administrated? Recommendation for passive immunisation are beyond the scope of this review, but details can be find elsewhere [6, 7].

## Which fundamental role does the primary care physician play

The primary care physician plays a critical role in optimising their patients’ protection against vaccine-preventable diseases. The first step is to identify within all their patients which are the ones who could benefit from an enhanced protection. When a child has a new diagnosis, and the immune system is likely to be affected, a quick review of the patient’s vaccination history should be automatic. The ascertainment of the patient’s protection status should rely on checking their records or serologies, as trusting oral recall only can lead to undervaccination or overimmunization [44]. Primary care physicians have a key role to play in discussing with families on the importance of vaccination and reassurance on their safety. In collaboration with a specialist team, a customised vaccination schedule could be planed and anticipated, aiming to immunise, for example, early in the disease process, anticipating periods of higher immunosuppression. This schedule should catch up missing vaccination and add the supplementary vaccines when needed. If recommended, vaccine seroresponses should be checked following vaccination and during follow-up visits.

Another fundamental role of the primary care physician is to ensure that all household members have their vaccinations updated. This “cocooning” strategy is a form of indirect protection for non-immune children, or for those who are unable to be vaccinated. Cocooning is, however, usually not sufficient to fully protect these children, especially those with normal lifestyles.

## Who are immune compromised children

Immunodeficiency can be primary or acquired, secondary to a disease, infection, medication, chronic organ failure or other state (e.g. malnutrition, young age) [8]. Medications can affect the immune system either as an undesirable side effect (e.g. chemotherapy, drug-induced neutropenia) or intentionally in conditions in which the immune response has to be restrained, e.g. management of autoimmune disorders and immune-mediated diseases, allergic disorders or solid organ transplant. The most common conditions encountered in daily practice are listed in Table 1.

**Table 1:** Summary of vaccine recommendations in children with chronic illness and/or immunosuppression

Medical condition	How is the immune system affected	Non-live vaccines recommendation	Live-attenuated vaccines recommendation ^a^	Additional vaccine(s) recommendation	Serological monitoring	Guidelines, references
Primary immunodeficiency disorders	Genetic abnormality affecting various pathway of the immune response	Routine ^b^	Permitted in certain situations only	IIV PCV (± PPSV23) MCV4 MenB if complement deficiency	“Regularly”, but no guidance on how often	ACIP [29] Reviews [43, 51–54]
Oncological diseases	Most cancers and their treatment affect the immune system	Routine during chemotherapy ^c^ Re-start vaccination as of 3m to 6m after completion of chemotherapy (including Hib, regardless of age)	Permitted as of 3m to 6m after completion of chemotherapy	IIV (even during chemotherapy) PCV (± PPSV23) MCV	No indication Could be useful to monitor seroprotection against measles and varicella	CCLG [48] IDSA [22] ACIP [29] AIEOP [55]
Hematopoietic stem-cell transplantation	Impaired and immature immune cells, loss of Ig	Revaccination starting 3m to 6m after HSCT (including Hib, regardless of age)	Revaccination permitted in certain condition as of 1.5y to 2y after HSCT	IIV PCV, 3d (± PPSV23) MCV, 2d	No indication Could be useful to monitor seroprotection against measles and varicella	CCLG [48] EBMT [49] IDSA [22] ACIP [29]
Solid organ transplantation	Immunosuppressive treatment	Accelerated schedule before SOT. Continue after SOT (as of 2m to 6m post-SOT)	Accelerated schedule if > 4w before SOT. Permitted in certain situation after SOT, as of 1y post-SOT [47]	IIV PCV (± PPSV23)	Frequent monitoring to guide vaccination; it can also inform on protection against measles and varicella	AST, IPTA [47] IDSA [22] ACIP [29]
Asplenia/hyposplenia Sickle cell disease	Higher risk of fulminant infection with encapsulated bacteria and parasite (highest risk in the first 2y of asplenia but persist life-long)	Routine, catch-up Hib vaccination regardless of age, HBV vaccination highly recommended if frequent transfusion. Anticipate 2w between vaccination and elective splenectomy	Permitted, as of a few days after splenectomy	IIV PCV (± PPSV23) MCV4 2d 2m apart, then every 5y MenB	Frequent monitoring of serotype-specific pneumococcal IgG to guide booster doses	IDSA [22] ACIP [29]
Human immunodeficiency virus infection	Lower CD4^+^ T-cell	Delay vaccination until viral load < 50 copies/mL and CD4 > 15% for 6m. Use high-dose HBV vaccine (40 μg) in adolescents. Give Hib vaccine regardless of age if not immune. DT booster at least 1×/10y.	Permitted only if CD4 > 200 cells/μl (or > 15–24% in infants and children) for > 6m	IIV PCV (± PPSV23) MCV4 2d 2m apart	Anti-HBs Ig periodically (if ongoing exposure) Anti-tetanus, anti-diphtheria 1×/5y Anti-measles, anti-rubella 1×/3–5y	PENTA [56] CHIVA IDSA [22] ACIP [29]
Immunosuppressive treatment for rheumatologic, renal, neurologic, gastrointestinal conditions	Underlying disease with dysregulated immune system, immunosuppressive treatment to control disease activity	Accelerate schedule before immunosuppression, but continue during and after	Permitted if low immunosuppression	IIV PCV (± PPSV23)	No indication but monitoring could guide booster doses and inform on protection, in particular against measles and varicella	IDSA [22] Review [16]
Complement inhibitors (eculizumab)	Medication inhibiting the deployment of the terminal complement system, high risk of meningococcal disease	Routine	Permitted	IIV PCV MCV4 MenB	No indication	Review [57]
Inflammatory bowel disease	Underlying defect in immune system, immunosuppressive treatment	Accelerate schedule before immunosuppression, but continue during and after	Permitted if low immunosuppression	IIV PCV (± PPSV23)	No indication, but monitoring could guide booster doses and inform on protection, in particular against measles and varicella	IDSA [22] Reviews [13, 16]
Nephrotic syndrome	Urinary loss of IgG, oedema, immunosuppressive treatment	Accelerate schedule before immunosuppression, but continue during and after	Permitted if low immunosuppression, VZV vaccine highly recommended	IIV PCV (± PPSV23)	Monitoring of serotype-specific pneumococcal antibody useful to guide booster. Monitor seroprotection against measles and varicella could be useful as well.	ACIP [29] Review [18]
Prematurity	Immune cell immaturity Low IgG level (not had time to transfer from the mother)	Accelerated schedule, based on chronological age	Accelerated schedule, based on chronological age	IIV PCV MCV RSV (cf country)	No indication	Review [58] AAP (RSV) [7]
Diabetes mellitus	Impaired phagocytic and neutrophil function, worsen with inadequate glycaemic control	Routine, HBV vaccination highly recommended	Permitted	IIV PCV (± PPSV23)	Documentation of protection against HBV. No other indication, antibody response to vaccinations seems to be normal overall	ACIP [29] Review [59] CDA (adults) [60]
Renal failure, chronic kidney disease (including dialysis)	Mild defects in T cell function, immune response impaired by various factor; Ig loss in dialysate	Accelerate schedule before dialysis, but continue during and after, HBV vaccination highly recommended	Permitted	IIV PCV (± PPSV23)	No indication, but monitoring could guide booster doses and inform on protection (vaccine responses likely to be impaired)	ACIP [29] Review [18]
Chronic liver disease	Impaired phagocyte function and defects in opsonising antibody, Ig loss in ascites, hyposplenism (with severe liver disease), higher risk of severe superimposed viral hepatitis	Routine, HAV and HBV vaccination highly recommended	Permitted	IIV PCV (± PPSV23)	No indication, but monitoring could guide booster doses and inform on protection	ACIP [29]
Chronic heart disease or malformation	Infections may precipitate cardiac decompensation	Routine	Permitted	IIV PCV (± PPSV23) RSV (cf country and underlying disease)	No indication	ACIP [29] AAP (RSV) [7]
Chronic lung disease Cystic fibrosis Bronchopulmonary dysplasia Asthma	Increased risk of severe respiratory infections. Severe lung diseases lead to poor mucociliary clearance, bronchiectasis, defects in pulmonary macrophage function	Routine	Permitted	IIV PCV (± PPSV23) RSV (cf country and underlying disease severity)	No indication	ACIP [29] AAP (RSV) [7]
Haemophilia	Historical increased risk of transfusion-related transmission of viral infection	Routine,^d^ HAV and HBV vaccination highly recommended	Permitted ^d^		No indication, adequate response to HBV vaccine could be documented	WFH [61]
Malnutrition Anorexia nervosa	Immune response impaired due to malnutrition	Routine	Permitted	IIV? *Insufficient data to date*	No indication	Review [62]
Obesity	Immune response slightly impaired due to overweight (and insulin resistance), higher risk of respiratory infection	Routine	Permitted	IIV	No indication, few studies reported lower vaccine responses	Reviews [63–65]
Coeliac disease	Functional hyposplenism (reversible), impaired immune response	Routine, HBV vaccination highly recommended	Permitted	IIV PCV (± PPSV23) ± MCV if hyposplenism confirmed	HBV serology (data suggest poor response to HBV vaccine administered prior to gluten-free diet)	Review [66, 67]
Chronic neurological disease and neurodevelopmental disorder	Decreased protection of airways increases risk of infection, higher risk of complication for some VPD (e.g. influenza, pneumococcus, varicella, pertussis)	Routine	Permitted, VZV vaccination highly recommended (higher risk of neurological complications)	IIV PCV	No indication	Recent article [68]
Inborn errors of metabolism	Neurological defect, concomitant immunodeficiency, metabolic decompensation	Routine	Permitted	IIV? PCV? *Insufficient data to date*	Unpredictable vaccine responses, depending on underlying immune defect	Review [21]
CNS anatomic barrier defect (e.g. CSF leak, inner ear dysplasia, or cochlear implant)	Deficient anatomical barrier leads to higher risk of CNS infection	Routine	Permitted	PCV (± PPSV23)	No indication	IDSA [22] ACIP [29]
Severe dermatologic conditions (severe eczema, psoriasis)	Chickenpox particularly prone to bacterial superinfection; severe dermatologic possibly require immunosuppressive treatment	Routine	Permitted if low immunosuppression, VZV vaccination highly recommended		No indication	Review [69]
Parents, close contact of immune compromised individuals	‘Cocooning’ strategy, to decrease the risk to transmit VPD to the immunocompromised children	Routine	Highly recommended if not immune, OPV and smallpox vaccine are the only LAV contra-indicated in close contact	IIV or LAIV	Documentation of immunity against measles and varicella if disease/vaccination history uncertain (or immunise regardless)	IDSA [22] Review [53]

The table summarises the vaccine recommendations available for various health conditions. Recommendations can differ between guidelines and between countries. In some countries, the cost of some vaccines may not be reimbursed. Recommendation for serological monitoring is rarely discussed in guidelines and the ones presented in this table summarise experts’ advices

^a^The live-attenuated influenza vaccine should never be given to immune compromised children as they can receive the inactivated influenza vaccine

^b^Effectiveness doubtful, depend on underlying disease and whether IVIG are given regularly

^c^Postpone if lymphocyte count < 1.0 × 10^9^/L. Non-live vaccine permitted during chemotherapy but will not be considered as “valid dose”

^d^Reduce the risk of bleeding by subcutaneous injection, use smallest gauge needle and applying pressure and/or ice for 3–5 min after injection

*AAP* American Academy of Paediatrics, *ACIP* Advisory Committee on Immunization Practices, *AIEOP* Italian Association Paediatric Haematology Oncology, *CDA* Canadian Diabetes Association, *CHIVA* Children’s HIV Association, *CSF* cerebrospinal fluid, *d* dose, *DTaP* diphtheria-tetanus-pertussis vaccine, *EBMT* European Society for Blood and Marrow Transplantation, *HAV* hepatitis A virus, *HBV* hepatitis B virus, *Hib Haemophilus influenzae* type b, *HSCT* hematopoietic stem cell transplantation, *IDSA* Infectious Disease Society of America, *Ig* immunoglobulin, *IIV* inactivated influenza vaccine, *IPTA* International Paediatric Transplant Association, *IPV* inactivated poliovirus vaccine, *IVIG* intravenous immunoglobulins, *LAIV* live-attenuated influenza vaccine, *LAV* live-attenuated vaccine, *m* month, *MCV* meningococcal conjugated vaccine, *MenB* meningococcus type B vaccine, *MMR* measles-mumps-rubella vaccine, *NLV* non-live vaccine, *OPV* oral polio vaccine, *PCV* pneumococcal conjugate vaccine, *PPSV23* 23-valent pneumococcal polysaccharide vaccine, *PENTA* Paediatric European Network for Treatment of AIDS, *RSV* respiratory syncytial virus, *SOT* solid organ transplantation, *VPD* vaccine-preventable disease, *VZV* varicella vaccine, *w* week, *WFH* World Federation of Hemophilia, *y* year

## Why are immune compromised children’s vaccination status not up-to-date

**Fig. 1:**
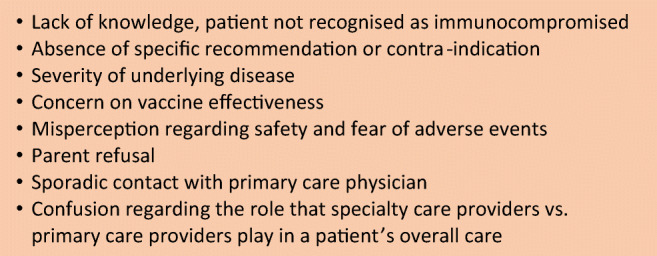
Common barriers to vaccination

**Fig. 2:**
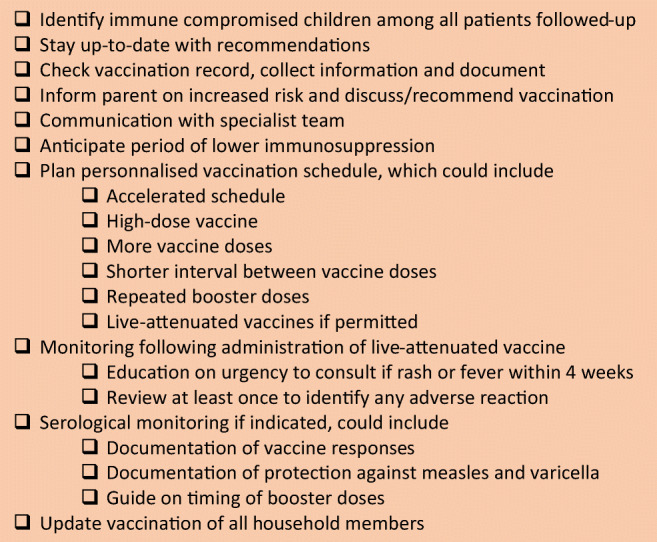
Checklist for primary care physician in optimising patients’ protection

Although vaccinations seem particularly indicated in this high-risk population, immune compromised children are often less adequately vaccinated than healthy children [9–11]. As vaccines and booster doses are given regularly throughout childhood, most children may not have completed their schedule before the onset of immunosuppression. But the main reasons underlying non-vaccination are summarised in Fig. 1 [11–13]. Moreover, as vaccination guidelines change frequently, and differ for each different medical condition, it is challenging to stay up-to-date with the most recent, specific recommendations [14]. As an example, it was recently reported in patients with inflammatory bowel disease (IBD) that vaccination was the least frequently followed quality of care recommendation [15]. In Italy, vaccination rates in children with HIV, cystic fibrosis, liver transplantation or diabetes were low against pneumococcus (< 25%) and highly variable for influenza (21% to 90%) [11]. Information and better communication appear to be key components for increasing vaccination uptake; primary care physician usually excels in both, being trusted by and close to the patient’s family (Fig. 2).

## Are vaccines immunogenic in immune compromised children

Concern on vaccine effectiveness is often an obstacle to vaccination in immune compromised children; immune response to vaccination can be suboptimal [16]. Vaccine responses may be reduced in both magnitude and durability, explaining the need for repeated monitoring of antibody levels during follow-up. However, even in highly immunocompromised hosts, vaccination may induce at least some immune response that could be beneficial in case of further encounter with the pathogen. Vaccines should therefore be administered despite possible non-responsiveness; in some medical conditions, monitoring of antibody concentration is recommended (Table 1).

## Are vaccines safe in immune compromised children

Whereas immunogenicity is an important aspect, vaccine safety is often the main concern of parents and healthcare practitioners. Live-attenuated vaccines (LAV), in particular, are usually avoided in the immunocompromised hosts as they could theoretically induce vaccine-strain infections; these are discussed in detail in a section below. In contrast, non-live vaccines are incapable of causing infection, since they consist in inactivated toxins (protein), in pathogen that have been killed (inactivated) or in only specific segments of the pathogen (subunit, polysaccharides) that may be conjugated to a protein (conjugate vaccine) to enhance the immunological response (Table 2). These vaccines can be given to immunocompromised patient without any safety concerns, as demonstrated in many studies in patients with chronic diseases, e.g. in HIV-infected individuals [17], patients with immune-mediated diseases [16], chronic kidney diseases [18] and solid organ transplantation recipients [19]. Therefore, information is critical to clearly explain the expected benefit to the patient.

**Table 2:** Summary of recommendation for vaccine administration and serological monitoring

Pathogen	Vaccine type	Vaccine recommendation	Rational for serological monitoring	Test used to measure seroprotection	Level required	Mechanism prevented
Diphtheria	Protein	Booster doses may be required more frequently; accelerated schedule in preterm or before onset of immunosuppression; effectiveness doubtful during cancer treatment and in children with primary immunodeficiency	Monitor vaccine response and guide for booster indication	Toxin neutralisation	0.01–0.1 IU/mL	Toxin production
Tetanus	Protein			Toxin neutralisation	0.1–0.1 IU/mL	Toxin production
Pertussis	Protein		No indication	ELISA	Not defined	Mucosal replication
Polio	Inactivated		Not routinely indicated	Serum neutralisation	1/4–1/8 dilution	Viremia
*Haemophilus influenzae* b	Conjugate	Catch-up regardless of age in some high-risk situation (hypo-/asplenia, HIV, after chemotherapy, after HSCT)	Could be used to document protection in high-risk situation	ELISA	1 ng/mL (polysaccharide) 0.15 ng/mL (conjugated)	Bacteraemia
Hepatitis A	Inactivated	Mainly recommended in travellers or if high risk of hepatitis	Not routinely indicated	ELISA	20 IU/L	Viremia
Hepatitis B	Subunit	Particularly recommended in cases of increased risk of needle-/transfusion-related transmission; supplementary vaccine doses and/or use of vaccine with higher antigenic dose may be required	Monitor vaccine response as poorly immunogenic in immunocompromised individuals	ELISA	10 IU/L (protective) >100–1000 IU/L (optimal)	Viremia
Human papillomavirus	Subunit	Strongly recommended in all immunocompromised conditions, with a 3-dose schedule regardless of age	No indication	ELISA	Not defined	Mucosal replication
Influenza	Inactivated	Recommended in all chronic diseases and immunocompromised conditions; clinical studies are ongoing to evaluate the need of high-dose vaccine in certain conditions	No indication	HAI	1/40 dilution (1/320 dilution in children)	Mucosal replication
Pneumococcus	Conjugate Polysaccharide	Recommended in all chronic diseases and immunocompromised conditions; indication for booster is less clear, mainly indicated in hypo-/asplenic patients	Could be used to guide for booster indication	Serotype-specific ELISA Serotype-specific OPA	0.35 μg/mL 1/8 dilution (differ among serotypes)	Bacteraemia
Meningococcus	Conjugate Polysaccharide	Mainly recommended when complement is affected, in oncological, HSCT, HIV-infected and hypo-/asplenic individuals, with a 2-doses schedule	No indication	ELISA Bactericidal test	2 μg/mL 1/4 dilution (human serum)	Bacteraemia
Measles	Live-attenuated	Accelerated schedule finishing at least 4 weeks before onset of immunosuppression. Permitted in some situations during immunosuppression (low immunosuppression, specific criteria for HIV and SOT)	Could be used to document protection in high risk situation	Microneutralisation assay ELISA	120 mIU/mL 150–200 mIU/mL	Viremia
Mumps	Live-attenuated		No indication	Serum neutralisation	Not defined	Viremia
Rubella	Live-attenuated		Could be used to document protection prior to pregnancy	Immunoprecipitation	10–15 mIU/mL	Viremia
Varicella	Live-attenuated	Accelerated schedule finishing at least 4 weeks before onset of immunosuppression. Permitted in some situations during immunosuppression (low immunosuppression, specific criteria for HIV and SOT). Highly recommended in some medical condition (e.g. nephrotic syndrome if low immunosuppression, neurological disorders, skin disorders)	Could be used to document protection in high risk situation	Serum neutralization Glycoprotein ELISA	1/64 dilution 5 IU/mL	Viremia

Adapted from [70–72]

*ELISA* enzyme-linked immunosorbent assay, *HAI* hemagglutination inhibition assay, *HIV* human immunodeficiency virus, *HSCT* hematopoietic stem cell transplantation, *OPA* opsonophagocytic assay, *SOT* solid organ transplant

Proper communication is particularly important for patients with immune-mediated diseases, inborn error of metabolism or solid organ transplantation, for which questions about the inflammation induced by vaccination could play a role in modulating the auto-immune response or inducing a metabolic crisis. Moreover, since the medical conditions of these children will be lifelong, vaccinations cannot be postponed indefinitely. Therefore, in these children, while it is important as for all children to monitor possible side effects of vaccination, the effect of the vaccine on the underlying condition should also be reported, such as signs of graft rejection or flare in disease activity. However, there is increasing data suggesting that concerns regarding the risk of disease exacerbation are unfounded, with numerous studies showing that immunization did not induce significant worsening of underlying disease [16, 19–21].

## Is the vaccination schedule the same than for healthy children

The vaccination schedules are usually the same; they slightly differ from those of healthy children in that they may include supplementary vaccinations (for example usually not given beyond a certain age), accelerated schedule, extra doses for primary vaccination, extra boosters, as well as specific conditions for administration of LAV. These are detailed in Tables 1 and 2, and in the following sections.

Recommendations are somewhat different between immunocompromising conditions: they are determined by the individual risk of infection and the data available. Among the various guidelines available, the Infectious Diseases Society of America provides a good overview of the current evidence available and covers most medical conditions (Table 1) [22]. The different national immunisation schedules can also be found online [23].

## Which supplementary non-live vaccines are indicated in which situation

Most immune compromised children benefit from protection against pneumococcus, influenza, meningococcus and human papilloma virus (HPV). These vaccines are included in many national guidelines for healthy children as well, so may not necessarily be considered as “supplementary vaccines”.

Invasive *pneumococcal* diseases carry a high mortality rate (11–30%) [24] and are more frequent in immunocompromised individuals, or those with chronic diseases, such as IBD [25], nephrotic syndrome [26] or a-/hyposplenic conditions [27, 28]. The pneumococcal conjugate vaccine (PCV) is usually recommended in healthy children before the age of 5, but also in all medical conditions with immunosuppression, regardless of age. Although some guidelines also recommend to subsequently administer the 23-valent polysaccharide vaccine (PPSV23) to those at high risk [29], many experts disagree, since PPSV23 do not induce memory cells and become less effective after repeated administrations (hyporesponsiveness) [30].

*Influenza*is probably the most common vaccine-preventable diseases leading to hospitalisation, accounting for 3.4% of all critical care admissions in the USA during the flu season [31]. In a retrospective cohort study in paediatric solid organ transplantation recipient, 40% of the hospitalisation for vaccine-preventable diseases were due to influenza infection [2]. Given the high burden of influenza disease, the vaccine is recommended in virtually all immune compromised children, as of 6 months of age. Moreover, preventing influenza also helps preventing secondary pneumococcal infection. Immune compromised children should always receive the inactivated vaccine and not the live-attenuated influenza vaccine (in Europe, the latter is only available in the UK).

*Meningococcal* vaccines are recommended to asplenic patients, HIV-infected individuals, those with complement deficiencies or receiving a treatment affecting the complement (such as eculizumab) [32]. .Most guidelines recommend a 2-dose schedule of the 4-valent conjugate vaccine (MCV4), and, when available, vaccination against serogroup B as well (Table 1).

As the risk of malignancy related to *HPV* is highly increased (up to 100-fold) in immunocompromised individuals [33], a 3-dose schedule is strongly recommended for all. The 2-doseschedule—used routinely in immunocompetent 11–15-year-old individuals—may not be sufficiently immunogenic, reason why the 3-dose schedule should be preferred [34].

## Do immune compromised children need more or higher doses

As vaccination may be less immunogenic in immune compromised children and immunity may wane faster, it is sometimes useful to administer vaccines with higher antigenic contents, additional vaccine doses or more frequent booster doses to ensure adequate response (via serological monitoring, as discussed below) and subsequent protection against vaccine-preventable diseases.

### High-dose vaccine

For vaccination against HBV per example, use of high-dose vaccine is recommended by some experts in HIV-infected adolescents (and adult), haemodialysis adult, and studies involving adults suggest it could be beneficial for oncological patients, or those with immune-mediated diseases [22]. Another example is the high-dose influenza vaccine being currently evaluated in immunocompromised individuals, including oncological patients, solid organ transplantation recipients and haemodialysis patients [35–37]. Data in paediatric patients, however, is scarce.

### More vaccine doses

Regarding schedule, 3-dose(rather than 2-doses) schedule are recommended for HPV in all immunocompromised condition, and a 2-dose (rather than single dose) schedule is recommended for MCV4 [22].

### More boosters

Regular MCV and PCV booster are recommended in some immunocompromised condition, whereas they are not recommended in healthy children. Diphtheria-tetanus booster doses are recommended more often as well, as guided by serological monitoring.

## The rationale behind serological monitoring

One of the most useful tools for customization of vaccination schedule in immune compromised children is to regularly monitor their serologies [38]. In children receiving chemotherapy for example, there is strong evidence to suggest that antibody concentrations wane more rapidly during treatment [39]. Cut-off values for seroprotection (i.e. correlates of protection) are available for most vaccine-preventable diseases (Table 2), but may vary slightly between laboratories. These measures allow to (i) confirm adequate vaccine response, (ii) guide when to administer a booster dose and (iii) document current protection against vaccine-preventable diseases. The latter is of particular importance for varicella and measles viruses, for which the reported mortality rates in infected immunocompromised hosts are up to 25% and 70%, respectively [3, 40, 41]. For both viruses, absence of seroprotection would require prompt management following contact (intravenous immunoglobulins and/or antiviral therapy), whereas documentation of highly seroprotective titres could suggest a “wait and see” attitude [42]. Physician can therefore inform individually on the risk of severe disease following contact with varicella or measles and provide guidance on what to do if this situation occurs. Regular monitoring of serologies against vaccine-preventable diseases has been adopted by many as an important part of the regular follow-up of immunocompromised patient. Although this test does not measure the other actors of the immune response, it is the only indirect measure of protection available. There is, however, no clear recommendation on when, in whom and how often should serology be assessed (Tables 1 and 2). Annual monitoring of serologies may be indicated in highly immunocompromised patients, or when the immunosuppressing regimen has recently been increased, whereas less frequent monitoring (i.e. once every 5 years) should probably be enough in well-controlled HIV-infected individuals, for example.

## In which situation can live-attenuated vaccines be administered

When vaccinating immunocompromised individuals, the most important safety issue concerns LAV. They consist in live pathogens that have been ‘weakened’ so that they can still replicate but with difficulty and without having the capacity to cause the disease in an immunocompetent host. Given the fear of a theoretical uncontrolled replication that could lead to severe vaccine-induced disease, LAV are mostly contraindicated in immune compromised children. In patients with severe primary immunodeficiency disease (e.g. severe combined immunodeficiency), LAV carry a significant risk of vaccine-strain infections, which have been reported following the oral rotavirus or poliovirus vaccines, measles-mumps-rubella(MMR) vaccine and bacille Calmette-Guérin vaccine [43, 44]. However, there is growing evidence documenting the safety of immunising immunocompromised hosts with different types of LAV in carefully selected settings.

MMR and varicella vaccines are usually well tolerated in case of milder immunosuppression, such as in children with DiGeorge syndrome (if lymphocyte count is > 500 cells/μL) [43], HIV-infected individuals (if CD4 count is > 200 cells/μL) [45, 46], liver or kidney transplant recipients (strict conditions [47]), after hematopoietic stem cell transplantation [48, 49], or in individuals with immune-mediated diseases on low/no immune suppression [16, 22], including children with nephrotic syndrome [50]. MMR and varicella vaccine have indeed the potential to protect patients against threatening pathogen that are endemic or linked with epidemics in many places around the world. However, extra caution should be taken and close safety monitoring is highly recommended following the administration of LAV in any situation when the immune system is affected [22, 47]. In the setting of solid organ transplantation, a consensus of worldwide experts has recommended the following surveillance: (i) education on urgency to seek medical attention in case of new onset of rash or fever within 4 weeks following vaccination and (ii) at least one contact with the patient’s caregiver in the month following vaccination to identify any adverse event that might have occurred [47].

## Concluding discussion

As many questions remain, clinical trials are still needed to refine the study of the immune response induced by each vaccine in all immunocompromising conditions to determine whether, when and for whom there is a need for a specific immunisation schedule. Moreover, additional guidance regarding the serological monitoring of vaccine response and persistence of protection is required.

As new vaccines become available and the epidemiology of vaccine-preventable diseases evolves, it is increasingly important for all those caring for children to be up to date with the recent changes to guidelines, in order to improve the usual low uptake of additional immunisations in high-risk groups [51]; physician‘s awareness is key, since it repeatedly correlates with higher vaccination rates [11].

## Data Availability

Not applicable.

## Abbreviations

IBD: Inflammatory bowel disease
HBV: Hepatitis B virus
HIV: Human immunodeficiency virus
HPV: Human papilloma virus
LAV: Live-attenuated vaccine
MCV: Meningococcal conjugate vaccine
MMR: Measles-mumps-rubella
PCV: Pneumococcal conjugate vaccine
RSV: Respiratory syncytial virus
